# Evaluation of the Immunostimulatory Effect of Ulvan Polysaccharide on Human Macrophages: Use as a Potential Vaccine Adjuvant

**DOI:** 10.3390/md23060248

**Published:** 2025-06-11

**Authors:** Valeska Guevara-Torrejón, Paola Chandía Parra, Carolina Campos-Estrada, Waleska E. Vera Quezada

**Affiliations:** 1 Laboratorio de Química de Metabolitos Bioactivos, Facultad de Farmacia, Universidad de Valparaíso, Valparaíso 2340000, Chile; vguevaratorrejon@gmail.com; 2 Laboratorio de Moléculas Bioactivas, Facultad de Ciencias del Mar, Universidad Católica del Norte, Coquimbo 1780000, Chile; nchandia@ucn.cl; 3 Laboratorio Toxicología, Facultad de Farmacia, Universidad de Valparaíso, Valparaíso 2340000, Chile; 4 Centro de Investigación, Desarrollo e Innovación de Productos Bioactivos, Universidad de Valparaíso, Valparaíso 2340000, Chile

**Keywords:** ulvan, sulphated polysaccharides, adjuvant, immunostimulant, vaccines

## Abstract

The ulvans are sulfated heteropolysaccharides that can stimulate the immune response in vitro. Using a human cell model, this study aimed to characterize and evaluate the immunostimulatory properties of crude ulvans extracted from *Ulva* spp., collected in Algarrobo, Chile. The crude ulvans, characterized by spectrophotometric methods, are composed of 47.6% total sugars, 14.3% uronic acids, and 8.9% sulfates, with an average molecular weight of 40.000 kDa. The FTIR spectrum showed bands related to uronic acids, rhamnose, and sulfate groups. GCMS analysis confirmed the presence of rhamnose, xylose, glucose, and galactose, with a predominance of the disaccharides U3s and B3s. HL60 cells differentiated into macrophages were cultured with three concentrations of crude ulvans (25, 50, and 100 μg/mL), with cell viability remaining above 90% at the lower concentrations. The crude ulvan activated CD86 co-stimulatory molecules and promoted the release of IL-6, IL-10, IL-4, and nitric oxide cytokines. The results suggest that ulvan is non-toxic and can activate inflammatory pathways, making it a potential candidate for studies as a vaccine adjuvant.

## 1. Introduction

Vaccines are a technological advance that exposes the immune system to an antigen to generate an immunological memory that allows it to recognize and fight it in case of a new exposure [1]. They are an efficient strategy to control and eradicate serious and even fatal diseases such as diphtheria, tetanus, yellow fever, pertussis, measles, smallpox, rubella, typhoid fever, and rabies, among others [2]. To reduce unfavorable reactions to vaccines, such as reactogenicity, vaccines incorporate an adjuvant to enhance and improve the immune response to an antigen [3]. In this way, adjuvants improve the quality of the immune response and can even reduce doses and inoculation frequencies in the population in some cases [4].

Different types of vaccine adjuvants are currently classified according to their composition and the mechanisms they interact with the immune system. The most known adjuvants and the first to be incorporated in vaccines are aluminum salts, used against infectious diseases such as tetanus, diphtheria, and hepatitis B [5]. There are also combinations of Monophosphoryl Lipid A (MPLA) with aluminum salts, such as Adjuvant System 04 (AS04), which has been used against human papillomavirus (HPV). Another formulation is Adjuvant System 01 (AS01), which is composed of MPLA and QS21 and has been used in research for vaccines against malaria, herpes virus, human immunodeficiency virus (HIV), and tuberculosis [6].

Commercially available adjuvants can enhance the immune response; however, more research is required to corroborate their long-term safety and understand their action method. In this sense, there is a need to search for new molecules of natural origin that can act as vaccine adjuvants.

Seaweeds vary in type and composition of bioactive molecules, highlighting the ulvans from green algae as potential immunostimulant molecules [7]. Ulvans are found in the cell wall of algae of the genus *Ulva* and belong to a family of structural sulfated heteropolysaccharides that are investigated for their potential uses as antihyperlipidemic, antioxidant, antitumor, anticoagulant, antiviral, and immunostimulant, among others [8].

**Figure 1:**
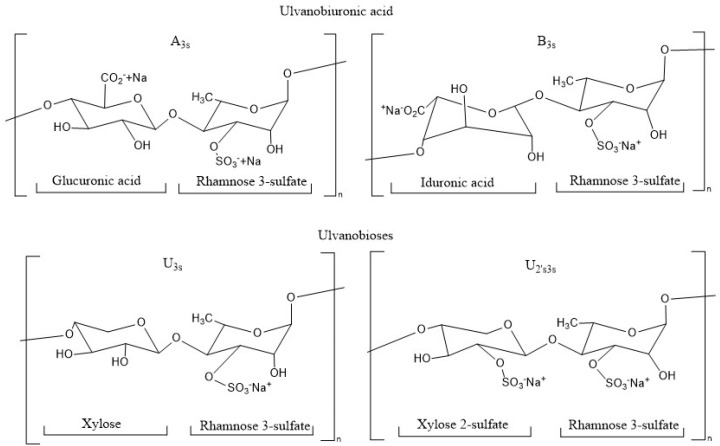
Chemical structure of repeating disaccharides in the ulvan structure.

The ulvans make up 8–29% of the dry weight of the algae [9] and consist of rhamnose units (up to 45%), xylose (2.1–12%), uronic acids such as glucuronic acid and iduronic acid (6.5–19%), together with sulfate-type functional groups (16–23.2%). From the structure of the ulvan chain, two disaccharides can be distinguished as repeating units of the ulvanobiuronic type and the ulvanobiosas. The ulvanobiuronics are differentiated into type A3s corresponding to α-L-sulfated rhamnose linked by the third non-reduced carbon with β-D-glucuronic acid and type B3s, which contains iduronic acid instead of glucuronic acid. As for ulvanobiosas, the U3s type consisting of β-D-xylose bound to the fourth carbon of sulfated L-rhamnose is identified, while the U2′s3s type consists of β-D-sulfated xylose bound to the fourth carbon of α-L-rhamnose 3-sulfate (Figure 1) [10].

The literature has recognized ulvans as polysaccharides with unique chemical compositions and interesting perspectives regarding possible immunostimulant activity. In their structure, glucuronic acids are identified, which are related to glycosaminoglycans of animal origin, such as hyaluronic acid and chondroitin sulfate, which have been reported as anti-inflammatory mediators capable of activating monocytes and B lymphocytes [11]. In addition, it highlights the presence of rhamnose in each of the monosaccharides that compose its structure, which is only found in plants and microorganisms and which can interact with protein receptors that regulate immunostimulatory activity [12], such as toll-like receptors (TLR) [13] and lectin receptors [14]. In this same line, sulfated polysaccharides are related to the stimulation and secretory activity of macrophages, in addition to the release of cytokines, chemokines, tumor necrosis factor (TNF-α), nitric oxide, and IL-1β, associating with an immunostimulatory effect.

Research on the evaluation of the immunostimulatory activity of ulvans has been conducted mainly in murine cell cultures, which report that the lower the concentration of ulvans, the higher the cell viability, indicating a non-cytotoxic concentration range of ulvans between 6.25 and 50 μg/mL [15,16]. Regarding cytokine secretion, it is indicated that cell cultures grown with ulvans promote an increase in the secretion of pro-inflammatory cytokines such as IL-1β, IL-6, and IL-12, and in turn, an increase in IL-10, which is anti-inflammatory, in which the authors relate it to the molecular weight and sulfate content of ulvan [12]. Other authors report the stimulation of an increase in the expression of pro- and anti-inflammatory mediators such as TNF-α, IL-6, and IL-10 [17], an increase in IL-1β in a dose-dependent relationship in lower molecular weight fractions of ulvans [16].

The immunostimulant action of ulvan depends on the structure and chemical composition of the polysaccharide, with essential factors being the species of algae, the locality, and the time of year it is extracted. In the commune of Algarrobo, Valparaíso, Chile, there are massive accumulations of algae of the *Ulva* genus, which have no commercial value and represent an interesting source of available biomass for chemical research and biological evaluation. This research aims to characterize the soluble polysaccharide from *Ulva* spp. and evaluate its immunostimulatory properties in a human macrophage cell model.

## 2. Results and Discussion

### 2.1. Extraction and Chemical Characterization of Ulvans Obtained

#### 2.1.1. Extraction and Spectrophotometric Chemical Characterization of Ulvans

From the pool of algae collected, the most abundant morphotype of algae of the genus *Ulva* spp. collected from Los Tubos de Algarrobo beach was selected and subjected to pretreatment and extraction with distilled water at 80–90 °C and neutral pH (≈7), which allowed an aqueous extraction aimed at obtaining the soluble polysaccharide, which resulted in a yield of 17% of the algal dry weight (Table 1), comparable to other studies where they performed a soluble polysaccharide extraction under the same conditions, which reported 17.8% for *U. pertusa*, 10–20% in *U. lactuca*, and 13% for *U. papenfussii* [18,19,20].

Polysaccharides are very complex, so characterization becomes indispensable to understanding and identifying their composition and chemical structure [21]. In this research, the aqueous polysaccharide extract obtained from *Ulva* spp. algae was characterized by three different methods: chemical characterization by spectrophotometric methods, spectroscopic methods such as FTIR, and chromatographic methods such as GCMS. The spectrophotometric analyses correspond to sugar constituents, uronic acids, sulfate groups, protein content, and numerical average molecular weight (Table 1) [22,23,24,25,26].

These techniques determined that the total sugar content in the crude ulvan was 47.6%, the sulfate content was 8.9%, and the uronic acid content was 14.3%, agreeing with that reported by other authors [9,27,28]. The uronic acids described for the crude ulvan obtained in this study refer to glucuronic acids and iduronic acids, which compose disaccharides of type A3S and B3S, respectively, grouped as ulvanobiuronic acids. They are associated with structures such as glycosaminoglycans that compose the extracellular matrix, such as heparin and chondroitin sulfate, possibly conferring the ulvan immunogenic properties desirable in regenerative medicine [29]. The sulfate content reported in this research coincides with that reported in other research [9]. However, it is essential to note that other studies have reported ranges that vary between 13 and 24% sulfate content, which have been obtained based on characterizations of soluble polysaccharides of different species of *Ulva* algae, in other places and times of the year [12], so this research broadens the range of sulfate content for algae of the genus *Ulva*. The number average molecular weight of the crude ulvans obtained was calculated based on the average of the reducing terminal groups of the fractions that make up the crude ulvan extracted from Algarrobo, resulting in a value of 40.000. This calculation does not have an associated unit of measurement, as it represents the relatively average size of the crude ulvan fractions in this investigation. This value can be interpreted as a crude ulvan from Algarrobo is a low molecular weight molecule, a favorable result for immunostimulatory evaluation where it is indicated that lower molecular weight polysaccharides promote a better immune response than those of longer chains [30,31]. As for the molecular weight of the ulvans, a significant variability has been reported [12,32] due to the different species of algae of this genus and the place of distribution in which the studies have been carried out [33]. Regarding protein analysis, it was impossible to detect proteins below 10 μg/mL by Bradford assay [25], which may mean that the extraction methodology was selective for soluble polysaccharides.

#### 2.1.2. Gas Chromatography Mass Spectrometry (GCMS)

After derivatization to alditol acetates, the identification of the monosaccharides L-rhamnose, D-xylose, D-glucose, and D-galactose that compose the structure of the crude ulvan extract obtained from the *Ulva* spp. algal pool was achieved.

**Figure 2:**
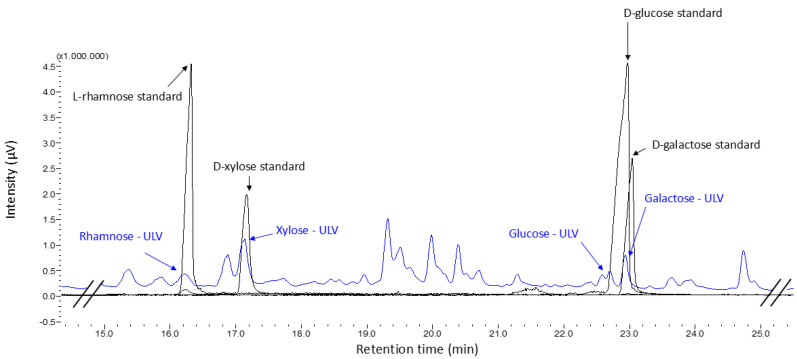
GCMS chromatogram of samples derivatized to alditol acetates. The standard monosaccharides are identified in black, and the peaks are labeled with their names. The crude ulvan sample is represented in blue, and the identified peaks are associated with the standards by their names—ULV.

The chromatographic signals of the monosaccharide standards were overlaid with the chromatogram obtained from the crude ulvan, with the signals for L-rhamnose, D-xylose, D-glucose, and D-galactose coinciding with the retention times presented by the crude ulvan sample, thus confirming the presence of these monosaccharides in the structure. These signals mainly align with fragments associated with ulvanobiuronic and ulvanobiose disaccharides (Figure 2). Additionally, through the analysis of the *m*/*z* spectra, we could propose the identification of the crude ulvan signals associated with the standards. The signal identified as sulfated rhamnose corresponds to *m*/*z* 191, hydrolyzed xylose to *m*/*z* 86, hydrolyzed glucose to *m*/*z* 115, and hydrolyzed galactose to *m*/*z* 150. Based on the interpretation of the chemical characterization and GCMS results, it is proposed that the crude ulvans in this study are predominantly composed of U3S-type ulvanobiose disaccharides, which consist of xylose linked to sulfated rhamnose. This is supported by the fact that the highest percentage of monosaccharide detected was xylose (48.9%), and rhamnose is the monosaccharide responsible for contributing sulfate to the molecule (Table 1). This coincides with that reported by other studies confirming that the extract obtained corresponds to ulvan [28,34].

#### 2.1.3. Fourier Transform Infrared Spectroscopy (FTIR)

**Figure 3:**
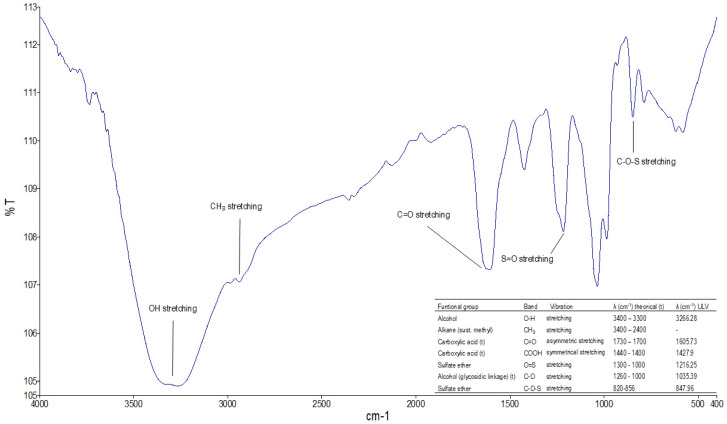
FTIR spectrum of crude ulvan obtained from algae of the *Ulva* spp. genus in the Valparaíso region.

FTIR characterization confirmed the presence of characteristic functional groups in ulvans extracted from *Ulva* (Figure 3). The bands obtained in the crude ulvan extract coincide with those described by other studies, such as those performed by [28,34,35], which are conducted from different species of *Ulva* spp. The broadband marked at 3266.28 cm^−1^ is attributed to the stretching vibrations of the O-H group associated with the uronic acids of the molecule. The approximate band at 2900 cm^−1^ is related to the stretching vibrations of the CH_3_ groups, related to the rhamnose molecules, the main monosaccharide that composes ulvan, and was confirmed by GCMS. The band at 1605.73 cm^−1^ is associated with asymmetric stretching vibrations of the carboxylic group associated with glucuronic and iduronic acid, which has been described in the disaccharide A3s and B3s of the ulvanobiuronic type. In addition, the band at 1427.9 cm^−1^ represents the bending vibration of the carbonyl group. The bands at 1216.25 cm^−1^ and 847.96 cm^−1^ indicate the S=O stretching vibrations of the sulfate ether and C-O-S of the sulfate ester in the axial position, respectively, confirming the presence of sulfate in ulvan [15,36]. The band at 1035.39 cm^−1^ is related to the stretching vibrations of the C-O group in rhamnose and the angle-shift vibrations of the O-H group that would form hydrogen bridges.

The above-mentioned background makes ulvans obtained from Algarrobo in the Valparaíso region an interesting candidate for immunostimulation studies in cultures in vitro.

### 2.2. Evaluation of the Inmunostimulant Effect in Cell Cultures

Research on the immunostimulatory effect of ulvans has been conducted mainly in murine cell cultures. While this coincides with many vaccines being tested in these models, the immune response differs due to differences in the expression of immune markers, such as the differential expression of TLRs [37]. Using a human promyelocytic leukemia HL60 cell line differentiated to macrophages is a strategy that allows understanding of the responses to the stimulation of the immune system, being this type of cell one of those involved as antigen presenters and interaction with T lymphocytes [38]; therefore, they are useful to study their cell differentiation, inflammation, and studies related to immune responses [39]. Based on this information, this research evaluated the cell viability of crude ulvan in human HL60 cell cultures differentiated into macrophages, followed by the immunostimulatory effect through cytokine release, CD86 surface marker activation, and nitric oxide release.

#### 2.2.1. Cell Viability

**Figure 4:**
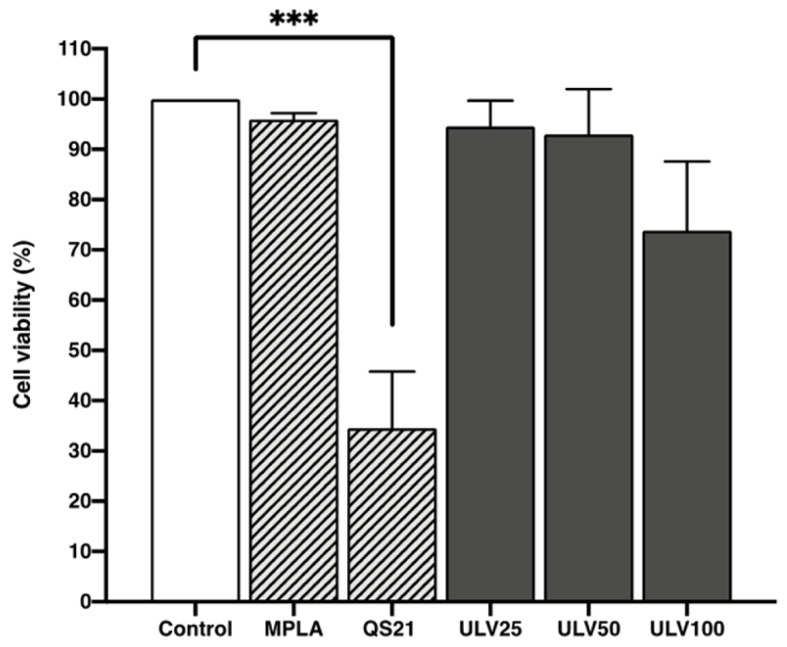
Cell viability assessment was done using the resazurin method in the HL60 cell line differentiated into macrophages incubated with different concentrations of vaccine adjuvants and crude ulvan. The data represent the mean ± standard deviation of four independent experiments. A one-way ANOVA analysis was performed. Statistical significance was defined as *** *p* > 0.01.

Ulvans have previously been reported to be non-cytotoxic to cell cultures, coinciding with the results obtained in this study (Figure 4), where ULV25 and ULV50 treatments resulted in viability more significant than 90% and ULV100 of 74%. In fact, in a study performed in murine macrophages, adding ulvan at 50 μg/mL promoted a 120% proliferation in cell culture [30]. The effect observed in the ULV100 treatment reached 74% viability. It can be related to a dose-dependent effect, where the higher the concentration of ulvan, the higher the cell culture mortality rate. As for the favorable control treatments, those cells cultured with MPLA resulted in viability higher than 90%, coincident with the control, which was expected since this is an approved adjuvant used in vaccines [40].

On the other hand, QS21 is an approved adjuvant for use as an AS01 formulation due to its reactivity and possible toxicity [41]. In this research, treatments with QS21 significantly reduced cell viability, reaching only 34%, which coincides with the results of other studies where dose-dependent toxicity is reported [42]. The three crude ulvan treatments achieve a higher viability than QS21, so it can be proposed as a less cytotoxic alternative that activates the immune system without causing cell death. Therefore, crude ulvan can respond without generating adverse effects as QS21.

#### 2.2.2. Indirect Inmunofluorescense

**Figure 5:**
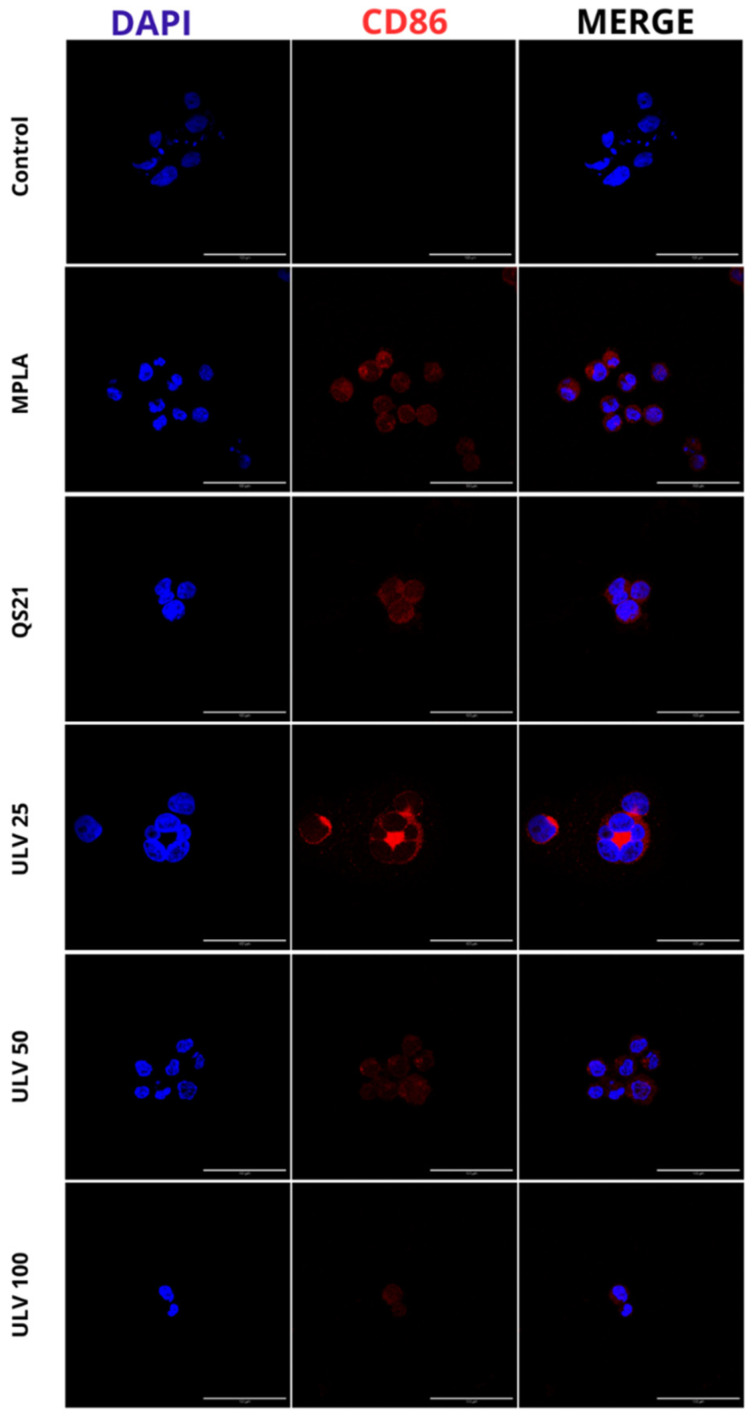
Qualitative analysis of CD86 protein expression in HL60 cell line differentiated into macrophages, incubated with different concentrations of vaccine adjuvants and crude ulvan. The indirect immunofluorescence method uses a primary antibody against CD86 and a secondary antibody, Alexa Fluor 647. The staining of membranes expressing CD86 protein in HL60 macrophage-differentiated cells is shown in red. The cell nuclei stained with DAPI are shown in blue.

Determining inflammatory markers in HL60 cell cultures differentiated to macrophages was performed by assessing the expression of CD86, a co-stimulatory molecule expressed on antigen-presenting cells. These cells initiate the cascade of immune system stimulation through regulatory T cells [43]. This is the first report of the immunostimulatory effects of crude ulvan using indirect immunofluorescence. Cell nuclei stained with DAPI are shown in blue, and red staining of the membranes of HL60 cells differentiated to macrophages confirms the expression of CD86 protein (Figure 5).

Through this technique, the differentiation to human macrophages of HL60 cells was confirmed since CD86 is expressed once macrophages differentiate into M1 and is absent in M0, which is a phenotype capable of promoting the expression of proinflammatory cytokines such as IL-6 and IL-1β [44]. In the presence of known effect adjuvants such as MPLA and QS21, a positive expression of this marker is observed, which has also been confirmed through flow cytometry in other cell cultures, confirming the activation of CD86 by commercial adjuvants such as MPLA and QS21 [18,45,46,47,48]. In turn, a positive signal is observed in all three concentrations of crude ulvan. The only condition that does not express this protein is the negative control, and it can be inferred that there is no inflammatory stimulus triggering a specific immune response or M1 in this condition.

#### 2.2.3. Cytokine Stimulation

**Figure 6:**
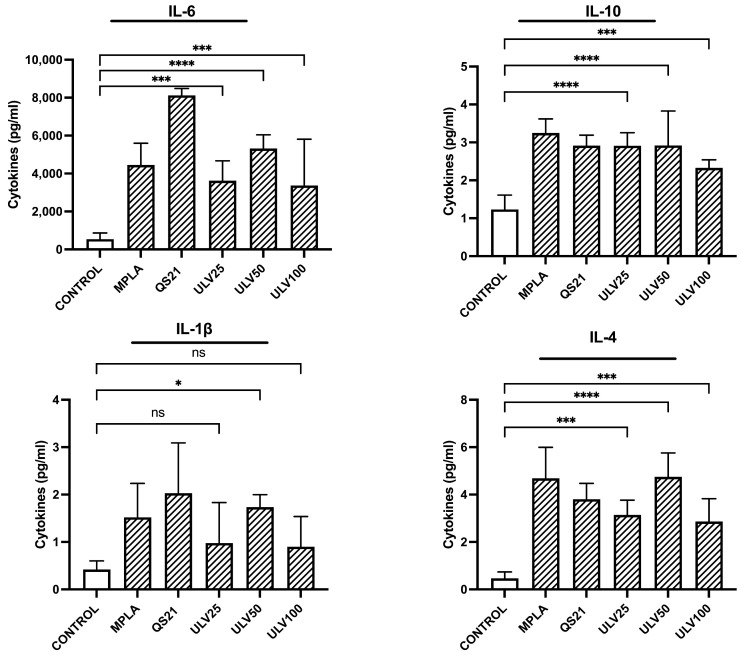
Cytokine expression in the HL60 cell line differentiated into macrophages, incubated with different concentrations of vaccine adjuvants and crude ulvan. The data represent the mean ± standard deviation of three independent experiments. Statistical significance was established with * *p* > 0.05; *** *p* < 0.01; **** *p* < 0.001; ns indicates not significant.

Ulvans have been reported to stimulate both proinflammatory and regulatory cytokines in cell cultures [30,31], a desirable feature for potential uses as a vaccine adjuvant. In this study, the secretion of cytokines with a pro-inflammatory profile, such as IL-6 and IL-1β, was evaluated, in addition to regulatory cytokines, such as IL-4 and IL-10 (Figure 6). All three crude ulvan treatments (25, 50, and 100 μg/mL) could significantly induce IL-6, IL-10, and IL-4 production compared to control, unlike IL-1β production, which only significantly increased secretion in ULV50 treatments. The cytokine IL-6 is regulated by CD86 activation [49] and, in the face of acute inflammation, acts as a proinflammatory; however, as the inflammatory stimulus is maintained over time, it exerts an anti-inflammatory role [50]. In the crude ulvan treatments performed in this study, ULV50 presented greater stimulation, followed by ULV25 and ULV100. The stimulation produced by ULV50 is comparable to that observed with MPLA. This is desirable since MPLA is a currently accepted and marketed vaccine adjuvant [51]; therefore, it is a good starting point for comparing possible effects. The treatment that stimulated the highest secretion was QS21, reaching 8000 pg/mL, demonstrating its potent stimulatory effect on the immune system; however, a high concentration of proinflammatory cytokines could trigger overexpression of inflammation, producing detrimental effects on the repair of damaged cells and tissues [52].

IL-1β is a cytokine induced by inflammatory signals. It acts as an amplifier of immune reactions, and in the crude ulvan treatments, ULV50 was the only one that presented a significant increase in control, as observed with the QS21 treatment. Other studies have evaluated IL-1β production in murine cell lines cultured in different concentrations of ulvan, indicating an increase in IL-1β but do not perform a more collected analysis regarding its modulation [16,30]. It has been reported that IL-1β can promote the differentiation of monocytes to dendritic cells and M1 macrophages [51]. The cytokine levels in this study support the inflammatory response of M1-type macrophages, concordant with the overexpression of IL-6 indicated in the previous paragraph.

About IL-4 secretion, this cytokine is categorized as regulatory. It has been studied to trigger the differentiation of cells to TH2 and the polarization of macrophages to M2 type, in addition to inducing the production of IL-10 [52]. This research indicates that the three crude ulvan conditions generate a higher IL-4 stimulation, with ULV50 presenting a very similar level to MPLA’s. In this sense, this result could provide relevant information on the possible effect of ulvans on the immune system. IL-10 is a key regulatory cytokine that prevents inflammatory and autoimmune pathologies [53]. It has also been reported to inhibit different inflammatory cytokine precursor complexes, such as IL-6 and IL-1β, up to CD80/CD86 co-stimulatory molecules [54]. In evaluating IL-10, all three crude ulvan treatments show a significant increase compared to the control, with ULV25 and ULV50 being the treatments with the highest significance. IL-10 stimulation is one of the desirable characteristics of ulvans, as it can counteract the effect of IL-6 and reestablish homeostasis in the immune response.

#### 2.2.4. Nitric Oxide

**Figure 7:**
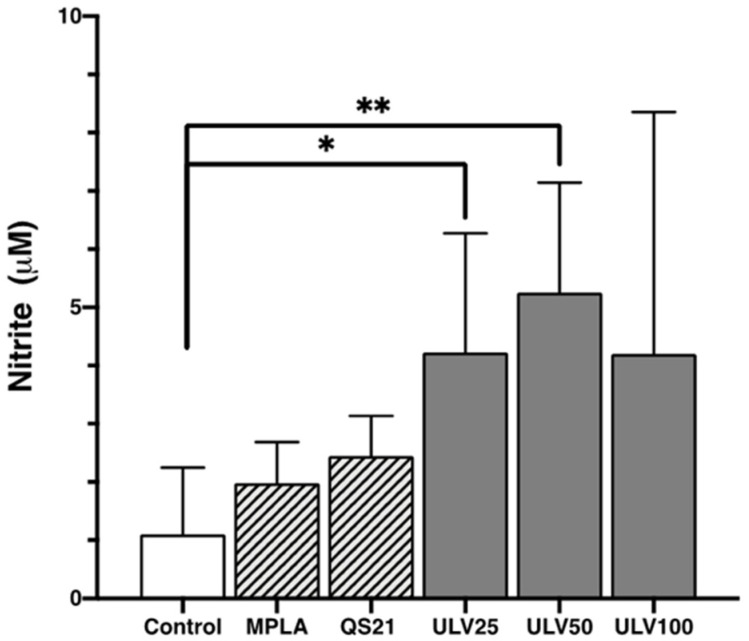
Nitric oxide expression in the HL60 cell line differentiated into macrophages, incubated with different concentrations of vaccine adjuvants and crude ulvan. The data represent the mean ± standard deviation of three independent experiments. Statistical significance was established with * *p* > 0.05; ** *p* > 0.01.

Nitric oxide released by cells indicates inflammation in M1-type cell culture [18], and nitrite release from macrophages in this study allowed for reaffirming the inflammatory response for all conditions experienced (Figure 7). The results indicate that ULV25 and ULV50 significantly increased nitrite release compared to the control, in which only ULV50 exceeded the release of 5 μM of nitrite. These results are comparable with cultured RAW 264.7 murine macrophages with ulvans at increasing concentrations of 10, 25, and 50 μg/mL, resulting in none of their concentrations exceeding 5 μM of nitric oxide [11]. Another study that cultured murine J774A.1 cells at different ulvan concentrations reported that the higher the concentration of ulvan, the higher the concentration of nitric oxide in the cultures, associating this with a dose-dependent effect [16]. However, with the highest concentration of ulvan (100 μg/mL), the cultures reached a concentration of 2.36 μM. In this study, all crude ulvan treatments released more than 4 μM of nitrite, which was not dose dependent since ULV50 values were higher than ULV25 and ULV100. These results may indicate that crude ulvan obtained from Algarrobo generates a more potent immune response, even than MPLA and QS21, which would position it as a potent immunostimulatory.

The secretion of pro- and anti-inflammatory molecules reported above hints at potential immunostimulant uses of vans. They may activate inflammatory signals that allow the immune system to react to a possible infection. At the same time, they may control this reaction by activating anti-inflammatory cytokines so that inflammation is not sustained over time. These functionalities of ulvans allow them to be projected as potential adjuvants for vaccines.

The importance of finding new molecules that can act as vaccine adjuvants adds to the immunostimulatory potential of crude ulvan, which is extracted from available biomass with no commercial value of *Ulva* spp. algae found in the locality of Algarrobo in the region of Valparaiso, Chile, generating an opportunity for the first report of immunostimulatory activity of crude ulvan from this locality.

## 3. Materials and Methods

### 3.1. Materials and Reactives

The pool of *Ulva* spp. algae was collected from Los Tubos de Algarrobo beach (33°21′50.5″ S 71°40′27.0″ W) on 22 October 2022 in the Valparaíso region, Chile. The algae were initially washed with potable water and then distilled water and finally dried in an oven at 50 °C, crushed, and stored in a dry and dark place [27]. The standards, L-rhamnose, D-galactose, D-glucose, and D-xylose, are from the Sigma^®^ brand. The cell line was HL60 human leukemia promyelocytic cells from ATCC (Manassas, VA, USA). These cells were differentiated for each experiment into human macrophages with 50 nM PMA.

### 3.2. Extraction of Ulvans

The dried and crushed algae were immersed in absolute ethanol for 24 h for pigment and lipid removal and then dried for 48 h at room temperature [16]. The extraction of ulvan was performed by immersing 100 g of pretreated dried algae in 3000 mL of distilled water in a thermoregulated bath at 90 °C for 3 h. After this, the algal material was filtered and subjected to a second extraction under the same conditions for 2 h. The supernatant obtained was centrifuged. The supernatant obtained was centrifuged for 10 min at 3500 rpm at room temperature. The supernatant was concentrated in a rotary evaporator at 50 °C to 1/3 of the total volume. The concentrated extract was purified by dialysis against distilled water for 72 h using dialysis membranes with a 3.5 kDa pore size. The dialyzed extract was again concentrated by rotary evaporator to 1/3 volume and then precipitated in a 3-fold volume of 95% ethanol. The precipitated extract was dried under a hood and then oven-dried at 50 °C until completely dry. The dried extract was then dissolved in distilled water to be freeze-dried for 15 days. The lyophilized extract of the soluble polysaccharide ulvan (crude ulvan) was stored in a dark and dry place until further use.

### 3.3. Chemical Characterization

#### 3.3.1. Spectrophotometric Chemical Characterization of Crude Ulvan

The quantification of total sugars was determined using the phenol-sulfuric method using glucose as a standard [22]. The uronic acid content was quantified using the m-hydroxydiphenyl method using glucuronic acid as a standard [26]. Sulfate content was analyzed using the turbidimetric method, with sodium sulfate as the standard [24]. Protein content was determined by the Bradford method using bovine serum albumin as standard [25]. Finally, the average molecular weight was calculated by quantifying reducing groups, using galactose as a standard [23]. In this case, a detection range of 1–13 μg/mL of galactose (n = 3) and a coefficient of variation of 0.2 have been used. Galactose is an epimer of glucose, sharing the same molecular weight. The only structural difference between them is the position of the hydroxyl group on carbon 4.

For the calculation of the number average molecular weight (Mn), the following equation was used:

(1) Mn=((Mmtra/Pr)∗180∗d

where,

Mn = number average molecular weight.
Mmtra = mass of the sample.
Pr = reducing power, expressed as the corresponding mass of the monosaccharide used in the calibration curve.
180 = constant, corresponding to the molecular weight of galactose.
d = correction factor for dilutions.

#### 3.3.2. Gas Chromatography Mass Spectrometry (GCMS)

For GCMS analysis, it was required to derivatize the crude ulvan extract to alditol acetates following the methodology of [55] with modifications. Then, 2.5 mg of crude ulvan was hydrolyzed with 0.5 mL of 2.0 M trifluoroacetic acid (TFA) in a glycerin bath for 2 h at 120 °C and the resulting solution was brought to dryness under reduced pressure using a rotary evaporator at 50 °C. The hydrolyzed sample was resuspended 10 times in 5 mL of distilled water and brought to dryness using a rotary evaporator again until pH 6 was achieved. It was then dissolved in 3 mL of distilled water with a spatula tip of sodium borohydride (NaBH_4_). This solution was left to react overnight, and then a spatula tip of Amberlite IR resin (H+) was added for 12 h. The solution was then removed and evaporated to dryness using a rotary evaporator at 50 °C; 500 μL of methanol was added to the balloon with the sample and taken to dryness, repeating the process 5 times. Subsequently, the balloons were stored in a vacuum desiccator overnight. The samples were redissolved in the minimum volume of anhydrous pyridine/acetic anhydride (1:1 *v*/*v*) and kept under constant agitation at 50 rpm for 24 h at room temperature. After this period, the solution evaporated under reduced pressure with successive ethanol additions. All standards were subjected to the same derivatization conditions as the crude ulvan to identify monosaccharides. The standards used were L-rhamnose, D-xylose, D-glucose, and D-galactose.

Crude ulvan and their respective derivatized standards were analyzed using a gas chromatograph coupled to a mass spectrometer (GCMS QP2010 Ultra, Shimadzu, Kyoto, Japan) following the methodology of [30] with modifications. An RXi-5ms capillary column (30 m × 0.25 mm id × 0.25 μm df) with helium as a carrier gas was used and maintained at a constant flow rate of 0.91 mL/min. Mass spectral acquisition was performed in the 35 to 500 *m*/*z* range, with full scan mode at a frequency of 1.56 scan/s, with electron impact ionization at 70 eV and a transfer line at 250°C. Chromatographic conditions were 160 to 210 °C for 10 min and then at 240 °C for 15 min, with a temperature gradient of 5 °C/min. One microliter of each sample diluted in chloroform was injected in splitless mode. Assignments and identification of the sample’s monosaccharides were performed by comparing retention times with independently injected authentic standards.

#### 3.3.3. Fourier Transform Infrared Spectroscopy (FTIR)

Potassium bromide (KBr) pellets were made using one measurement of crude ulvan and 2 measurements of KBr, which were mixed using an agate mortar. The solids were then pressed until a uniform pellet was formed. The pellet was analyzed using a Spectrum UATR two PerkinElmer model FTIR spectrum with 4000 cm^−1^ and 400 cm^−1^ measurements. Sixty-four scans were performed with a resolution of 2 cm^−1^ [56].

### 3.4. Evaluation of the Inmunostimulant Effect in Cell Cultures

For all experiments in this section, HL60 cells were cultured in a well pretreated with Matrigel^®^ (1/100 dilution) and differentiated to human macrophages using 2 μL of 50 nN PMA diluted in 200 μL of RPMI 1640 culture medium supplemented with L-glutamine, 10% heat-inactivated fetal bovine serum, and 1% penicillin/streptomycin. The plate was incubated in an incubation chamber for 48 h at 37 °C with humidity and 5% CO_2_.

After that, HL60 cells were differentiated into macrophages and cultured for 24 h in experimental conditions indicated in Table 2. After 24 h, the supernatant of each experimental condition was removed, and the immunostimulatory activity was evaluated through three experiments.

#### 3.4.1. Cell Viability

Cell viability was determined using the Resazurin method [57]. Each well was seeded with 3 × 10^4^ differentiated HL60 cells previously incubated with the experimental conditions. One hundred and fifty microliters of Resazurin was added to each well at a final concentration of 44 µM and incubated for 4 h at 37 °C in the dark. Finally, cell viability was determined by plate reading using fluorescence at 560/590 nm.

#### 3.4.2. Indirect Inmunofluorescense (IFI)

The activation of the surface marker CD86 in HL60 cells differentiated to human macrophages using an IFI assay was used as a model to determine the inflammatory processes. In addition to the above preparations, for this experiment, the 24-well flat-bottom plate had to be conditioned with a round coverslip previously sterilized and subsequently conditioned with Matrigel^®^ (1/100 dilution). Differentiated 2 × 10^5^ HL60 cells were cultured for 24 h under the experimental conditions indicated in the previous section. After this time, the IFI protocol was performed according to Thermo Fisher Scientific© (Waltham, MA, USA) manufacturer’s specifications. The plate was worked on a bed of ice, and the supernatant was removed to add 250 μL of 4% PFA for 20 min to fix the cells. Subsequently, the plate was washed with 0.1 M phosphate-buffered saline (PBS) at pH 7.4 for 5 min, the supernatant was removed, and the cells were permeabilized with cold methanol for 20 min. After permeabilization, they were rewashed with 0.1 M PBS and incubated for 1 h with a blocking solution composed of normal goat serum, 0.1 M PBS, the nonionic surfactant Triton X-100, and bovine serum albumin. The plate was then cultured with CD86 primary antibody (1/100) at room temperature for 4 h. After that time, the supernatant was removed, washed with 0.1 M PBS for 5 min, and Alexa Fluor 647 secondary antibody (1/500) was added. The plate was protected from light and incubated at room temperature under constant 50 rpm agitation for 1 h. Finally, the nuclei were stained with DAPI (2 µg/mL) for 5 min and washed with 0.1 M PBS for the last time. The coverslips with the cells were mounted and fixed on a slide and then photographed with a Nikon Eclipse S2 epifluorescence microscope.

#### 3.4.3. Cytokine Stimulation

Supernatants from cell cultures were incubated under experimental conditions and stored since the Resazurin assay was analyzed. The analysis was performed through a multiplex assay using Luminex^®^ Multiplex Assays technology, following the manufacturer’s instructions. This methodology determines the expression of the following cytokines: IL-1β, IL-4, IL-6, and IL-10.

#### 3.4.4. Nitric Oxide

NO release in cell culture was measured using the Griess reaction [58]. Approximately 3 × 10^4^ HL60 cells differentiated into macrophages were cultured in 96-well flat-bottom culture plates under previously used conditions. After the time had elapsed, 100 μL of the supernatant of the cultured cells was reacted with an equal volume of Griess reagent (1%, *w*/*w* vol) and incubated at room temperature for 10 min. The absorbance was measured at 540 nm with a microplate reader. NO production from macrophages was compared against a standard curve obtained with NaNO_2_ (1–200 μM in culture medium).

### 3.5. Stadistical Analysis

All cell cultures were performed in triplicate with n = 3 for each independent experiment. Data obtained are expressed as the mean or percentage ± standard deviation of cultures per triplicate. For data with normal distribution, parametric analyses and two-way ANOVA analysis of variance with Tukey’s test for multiple comparisons were performed, considering for this a significance level of *p* < 0.05. In addition, image processing was performed using the ImageJ-win64 digital processing program, and the results were analyzed using the GraphPad Prism 8.0^®^ statistical program.

## 4. Conclusions

Based on the results obtained, it can be confirmed that the polysaccharides extracted from algae of the genus *Ulva* spp. collected from the Valparaíso region correspond to crude ulvan, which has a predominant chemical composition of disaccharides of the ulvanobiose type U3s, with an average numerical molecular weight of 40,000.

As for their evaluation, crude ulvan did not generate cytotoxic effects. The results indicate that they can induce the expression of co-stimulatory molecules such as CD86 that trigger inflammatory processes through IL-6 cytokines, which are then controlled by regulatory cytokines such as IL-10. In addition, it promotes the release of nitric oxide, which is also an indicator of immunostimulation of the immune system. Of the three concentrations of crude ulvan evaluated in this study, it is proposed that the concentration of 50 μg/mL presented the best immunostimulatory effects, which were also concordant with those obtained with the approved commercial adjuvant MPLA, suggesting a similar immunostimulatory action.

This is the first report on the characterization of the crude ulvan in the Valparaíso region, positioning Los Tubos de Algarrobo beach as a possible source of available biomass. The chemical characterization carried out in this research can be the basis for further research related to the chemical composition of the ulvan at different times of the year. The effect of the studied crude ulvan opens a research opportunity related to the immunostimulatory effect with perspectives and projections to a possible formulation as a vaccine adjuvant based on extracts obtained from waste algae, aiming to solve a current problem regarding the accumulation of green algae in tourist coastal localities.

## Figures and Tables

**Table 1:** Characterization of ULV extracted from *Ulva* spp. algae pool.

	Crude Ulvan
**Composition**	
Yield (%)	17
Total sugars (%)	47.6
Uronics acids (%)	14.3
Protein (%)	ND ^1^
Sulfate (%)	8.9
**Monosaccharide composition**	
Rhamnose (%)	19.6
Xylose (%)	48.9
Glucose (%)	4.4
Galactose (%)	27.2

ND ^1^: not detected.

**Table 2:** Experimental conditions were used for evaluations in HL60 cell line that were differentiated to human macrophages.

Experimental Design		
Treatment	Abbreviation	Concentration
Culture medium	MC	RPMI 1640, supplemented with L-glutamine, 2% heat-inactivated fetal bovine serum, and 1% penicillin/streptomycin.
Monophosphoril Lipid A	MPLA	2 μg/mL in MC
QS21	QS21	1 μg/mL in MC
Crude ulvan 25	ULV25	25 μg/mL in MC
Crude ulvan 50	ULV50	50 μg/mL in MC
Crude ulvan 100	ULV100	100 μg/mL in MC

## Data Availability

The original data presented in the study are included in the article; further inquiries can be directed to the correspondings authors.

## Abbreviations

The following abbreviations are used in this manuscript:

MDPI	Multidisciplinary Digital Publishing Institute
DOAJ	Directory of open access journals
ULV	Ulvan
MPLA	Monophosphoryl Lipid A
AS04	Adjuvant System 04
AS04	Adjuvant System 01
HPV	Papillomavirus
HIV	Human immunodeficiency virus
TNF-α	Tumor necrosis factor
IL-1β	Interleukin IL-1β
IL-6	Interleukin IL-6
IL-4	Interleukin IL-4
IL-10	Interleukin IL-10
FTIR	Fourier transform infrared spectroscopy
GCMS	Gas chromatography mass spectrometry
IFI	Indirect immunofluorescence
CD86	Co-stimulatory molecule
